# A Model of Chronic Temporal Lobe Epilepsy Presenting Constantly Rhythmic and Robust Spontaneous Seizures, Co-morbidities and Hippocampal Neuropathology

**DOI:** 10.14336/AD.2019.0720

**Published:** 2019-10-01

**Authors:** Dinesh Upadhya, Maheedhar Kodali, Daniel Gitai, Olagide W Castro, Gabriele Zanirati, Raghavendra Upadhya, Sahithi Attaluri, Eeshika Mitra, Bing Shuai, Bharathi Hattiangady, Ashok K Shetty

**Affiliations:** Institute for Regenerative Medicine, Department of Molecular and Cellular Medicine, Texas A&M Health Science Center College of Medicine, College Station, TX, USA

**Keywords:** chronic epilepsy, cognitive and mood dysfunction, EEG recordings, inhibitory interneurons, spontaneous recurrent seizures, temporal lobe epilepsy

## Abstract

Many animal prototypes illustrating the various attributes of human temporal lobe epilepsy (TLE) are available. These models have been invaluable for comprehending multiple epileptogenic processes, modifications in electrophysiological properties, neuronal hyperexcitability, neurodegeneration, neural plasticity, and chronic neuroinflammation in TLE. Some models have also uncovered the efficacy of new antiepileptic drugs or biologics for alleviating epileptogenesis, cognitive impairments, or spontaneous recurrent seizures (SRS). Nonetheless, the suitability of these models for testing candidate therapeutics in conditions such as chronic TLE is debatable because of a lower frequency of SRS and an inconsistent pattern of SRS activity over days, weeks or months. An ideal prototype of chronic TLE for investigating novel therapeutics would need to display a large number of SRS with a dependable frequency and severity and related co-morbidities. This study presents a new kainic acid (KA) model of chronic TLE generated through induction of status epilepticus (SE) in 6-8 weeks old male F344 rats. A rigorous characterization in the chronic epilepsy period validated that the animal prototype mimicked the most salient features of robust chronic TLE. Animals displayed a constant frequency and intensity of SRS across weeks and months in the 5th and 6th month after SE, as well as cognitive and mood impairments. Moreover, SRS frequency displayed a rhythmic pattern with 24-hour periodicity and a consistently higher number of SRS in the daylight period. Besides, the model showed many neuropathological features of chronic TLE, which include a partial loss of inhibitory interneurons, reduced neurogenesis with persistent aberrant migration of newly born neurons, chronic neuroinflammation typified by hypertrophied astrocytes and rod-shaped microglia, and a significant aberrant mossy fiber sprouting in the hippocampus. This consistent chronic seizure model is ideal for investigating the efficacy of various antiepileptic drugs and biologics as well as understanding multiple pathophysiological mechanisms underlying chronic epilepsy.

Epilepsy, a major neurological disorder after stroke, affects 3 million Americans and ~50 million people worldwide [1]. Temporal lobe epilepsy (TLE), the most common type of epilepsy, is characterized by the progressive development of complex partial seizures, hippocampal neurodegeneration, and co-morbidities such as cognitive and mood impairments [2-4]. Numerous animal prototypes of epilepsy have been developed for understanding epileptogenesis, modifications in electro-physiological properties, neuronal hyper-excitability, neurodegeneration, neural plasticity, chronic neuro-inflammation in TLE [5-7]. The most commonly employed models of TLE include maximal electroshock seizures (MES), kindling-induced seizures or injections of chemoconvulsant compounds such as pentylenetetrazole (PTZ), pilocarpine or kainic acid (KA). Administration of chemoconvulsant drugs induces status epilepticus (SE), which evolves with time into TLE, typified by spontaneous recurrent seizures (SRS) [5]. Many of these models have been extensively used to study multiple epileptogenic alterations, loss of excitatory and inhibitory interneurons, changes in electrophysiological properties of neurons and astrocytes, persistent oxidative stress, chronic neuroinflammation, cognitive and mood impairments in TLE, and to test the efficacy of AEDs, cell therapy, gene therapy or other biologics for easing SRS. However, consistency in the frequency, pattern, and severity of SRS, and the manifestation of co-morbidities in these models have been a subject of intense discussion [5, 8-10]. An ideal model of chronic TLE would need to display stability in SRS frequency, the pattern of seizure occurrence in daylight and night periods, the intensity and severity of seizures, and the manifestation of cognitive, memory and mood impairments. Such a model would facilitate efficient pre-clinical testing of candidate therapeutics for easing SRS and the related co-morbidities.

The KA model of TLE has been considered as a highly isomorphic model of the human disease, independently of the method of KA administration [11]. Several studies have employed administration of KA through different routes for studying multiple features of TLE. Kainic acid is a potent excitatory cyclic analog of L-glutamate and an agonist of the ionotropic KA receptor. Systemic, intracerebral, or intracerebroventricular administration of KA causes neuronal depolarization and seizures, with multiple pathological changes preferentially targeting the hippocampus [12-21]. In the initial studies, intra-amygdaloid injection of kainic acid was used to develop epileptogenic changes and repetitive secondarily generalized convulsive seizures [22-23]. An intrahippocampal injection of KA caused acute effects and a silent phase followed by a period with SRS [24] while single dose systemic administration of KA also altered behavioral and electrographic activities [25]. Unilateral intracerebroventricular KA administration at a low dose (0.5 µg) induced consistently robust neurodegeneration in the CA3 pyramidal cell layer and partial neurodegeneration in the dentate hilus and the CA1 pyramidal cell layer in the hippocampus ipsilateral to KA administration with negligible mortality [13-14, 16-21, 26]. In addition, this model, showed consistent epileptogenic changes in the hippocampus, which include DG and CA1 hyperexcitability associated with loss of significant numbers of interneurons [27-30], aberrant mossy fiber sprouting [19, 21, 28], sprouting of entorhinal axons [31], abnormal and increased neurogenesis in the early phase and considerably waned neurogenesis in the chronic phase [32]. However, the major limitation of this milder model of TLE was the frequency of SRS, which was minimal and was seen at extended time-points after ICV KA administration.

Hellier and associates utilized systemic KA administration with several modifications to understand epileptogenic processes and to develop a robust model of TLE displaying significant SRS in the chronic phase using Sprague-Dawley (SD) rats [33-34]. Hourly systemic administration of low doses of KA for several hours in SD rats induced recurrent motor seizures for ≥3 hours with a relatively low mortality rate of 15%, which eventually led to a chronic epileptic state after a latent period [33-34]. Rao and colleagues extended Hellier protocol [33] to the Fischer 344 (F344) strain where 4-5 months old male rats were injected intraperitoneally with KA at 3 mg/Kg/hour for 4 hours [35-39]. This induced intense status epilepticus with bilateral neurodegeneration in different regions of the hippocampus and extrahippocampal regions. Also, in the chronic phase after SE, animals displayed robust SRS (~2.6/hour) associated with considerable aberrant mossy fiber sprouting, loss of interneurons and considerably waned neurogenesis [35-39], However, the mortality rate was higher (30-40%) in this model as continuous seizure activity observed for 3-4 hours after SE was not terminated with diazepam. Another study utilizing a single 10 mg/Kg dose of intraperitoneal KA demonstrated lower mortality but considerable heterogeneity in SRS onset and frequency [40]. Only 26% of animals exhibited robust seizures (>4 seizures/day) while the remaining animals showed greatly diminished frequency of SRS (~0.5 seizure/day). Recently, Bertoglio and colleagues compared KA-induced SE between Wistar Han (WH) and SD rats using two distinct protocols [41]. With the Hellier protocol [27], WH rats showed higher mortality than SD rats. When an initial dose of KA was increased to 7.5 mg/Kg followed by repeated low-doses (2.5mg/kg) every 30 minutes, similar severity of SE with better survival was observed in both strains. Interestingly, in comparison to WH rats, SD rats displayed a much shorter latent phase to develop chronic epilepsy and a higher frequency of SRS/day, despite having reduced neurodegeneration than WH rats [41]. Another recent study elicited focal hippocampal seizures for a few hours in SD rats through coincident subcutaneous injections of KA (~15 mg/Kg) and lorazepam (0.75 mg/Kg) with 0% mortality [42]. This model demonstrated spontaneous focal hippocampal seizures starting ~12 days after the administration of KA and lorazepam with classic hippocampal sclerosis and mossy fiber sprouting but no convulsive seizures. Moreover, KA-induced SE in C57BL6/J mice resulted in chronic epilepsy, but spontaneous convulsive seizures peaked at 4-6 weeks post-SE and decreased subsequently [43]. In summary, while the above animal prototypes are useful for addressing specific questions related to TLE, an animal prototype of chronic TLE displaying consistent convulsive SRS over days, weeks, and months with the manifestation of co-morbidities is yet to be validated.

The current study focused on developing a KA model of chronic bilateral TLE with minimal mortality and constantly rhythmic and robust SRS with comorbidities. We employed 2-5 graded intraperitoneal injections of KA at 5 mg/Kg to young F344 rats to induce SE and terminated convulsive seizures through a dose of diazepam at two hours after the onset of SE. This approach resulted in a prototype of TLE presenting minimal mortality during the SE phase (<10%) and a robust SRS in the chronic phase in a vast majority of animals (>90% of KA-injected animals). Through continuous EEG recordings, we demonstrate that this model offers consistent frequency and intensity of SRS per day over 21 days in both 5^th^ and 6^th^ month after SE. Furthermore, analyses of the 24-hour periodicity of SRS over 21 days in both 5^th^ and 6^th^ month after SE demonstrated consistently rhythmic SRS with the majority of SRS occurring in the light phase when the animals were less active. These epileptic animals also showed impaired cognitive, and memory function and depressive-like behavior. Overall, an intraperitoneal KA prototype of TLE described in this study is ideal for testing AEDs or biologics having promise for easing SRS and comorbidities.

## MATERIALS AND METHODS

### Animals

Young adult (6-8 weeks old) male F344 rats, obtained from Harlan, were used in this study. A total of 193 rats were used in the study. Animals, housed in an environmentally controlled room with a 12:12-hr light-dark cycle, received ad libitum food and water. All animal experiments performed in this study have been approved by the institutional animal care and use committee of the Texas A&M University.

### Induction and termination of SE

After 7-10 days of familiarization in the vivarium, SE was generated in 173 rats in multiple batches. Animals in each batch, comprising 9-12 rats, received graded intraperitoneal injections of KA at 5?mg/Kg every hour for 2-5 hours until they exhibited either a state of unremitting stage IV seizures exemplified by bilateral forelimb clonus with indications of rearing, or a first stage V seizure displaying bilateral forelimb clonus with rearing and falling. Rats continued to have multiple stages III-V seizures for two hours after the onset of SE. The behavioral seizures were terminated at 2 hours after the beginning of first stage IV/V seizure through subcutaneous diazepam (10?mg/Kg) injection.

### Recording of behavioral seizures

Two months after the induction of SE, animals in all batches that developed SE and survived were carefully examined for the occurrence of behavioral SRS through direct observations. The behavioral seizure scoring was performed in 6-hour sessions for 8 days, for a total of 48 hours. The parameters such as the frequency of all SRS, the frequency of stage V-SRS (the severe form of SRS), the average duration of individual SRS and the total time spent in SRS activity were measured. The scoring of behavioral SRS was done to confirm the development of chronic epilepsy after SE. Animals that showed repeated SRS were designated as chronically epileptic rats (CERs).

### EEG electrode implantation surgery and recordings of EEG

Two cohorts of CERs (6-12 rats/cohort) were implanted with EEG electrodes to measure the frequency, intensity, and consistency of SRS over many weeks, using 24/7 video-EEG recordings from a time-locked video-digital EEG monitoring system (AS40 from Grass-Telefactor), in the 5^th^ and 6^th^ month after SE. The methods employed for implantation of EEG electrodes are detailed elsewhere [35]. In brief, each rat was deeply anesthetized and fixed to a stereotactic device. Burr holes were made in the skull, and three sterile metal EEG recording electrodes with mounting screws were implanted epidurally, which comprised two recording electrodes over the frontoparietal cortices (one on each hemisphere), and a reference electrode over the cerebellum. A few screws without wires were also placed over the frontal cortex for holding dental cement. The electrode leads were attached to a micro plug, following which all electrodes and screws were cemented to the animal's head. Two weeks after the EEG implantation surgery, each rat was placed in a Plexiglas cage and connected to a tethered video-EEG system for continuous EEG recordings. The electrode pedestal on the rat's head was connected to the video-EEG system through a connector cable to continuously monitor both behavior and electrographic activity in freely behaving animals with continuous access to food and water. In each rat belonging to 2 cohorts, EEG was recorded continuously for 21 days. The EEG tracings were rigorously analyzed for the frequency of all SRS, the frequency of stage V-SRS, the average duration of individual seizures, and the total time spent in seizure activity. The entire seizure data were further stratified to evaluate diurnal variations.

### Analysis of the temporal pattern of SRS occurrence

The number of SRS per 1-hour time bin was scored for rats in each cohort. The analysis of 24 hour-distribution of SRS was performed by using Acrophase software, which detected the 24 hour-periodicity through a cosinor regression and calculated the acrophase of temporal distribution with the 95% confidence interval. This analysis was done separately for each animal and the total seizures among all animals in each cohort.

### Evaluation of co-morbidities in CERs

We examined a larger cohort of CERs (n=20) with many behavioral tests at four months after SE and compared the results with age-matched naïve control rats (n=20) to discern cognitive and mood dysfunction. The behavioral tests comprised a hippocampus-dependent cognitive test (an object location test, OLT), the perirhinal cortex and hippocampus function related recognition memory test (a novel object recognition test, NORT), the dentate gyrus function-dependent pattern separation test (PST), and a test measuring anhedonia (a sucrose preference test, SPT). The object-based tests (OLT, NORT, and PST) were performed using an open field apparatus measuring 100 cm x 100 cm.

### Object location test (OLT)

Each rat explored an open field with three sequential trials separated by 15-minute inter-trial intervals, as described in our previous reports [44-45]. In succinct, the rat was placed in an open field with no objects in the first trial for familiarization to the testing apparatus for 5 minutes (the habituation phase) whereas, in trial 2, the rat was allowed to explore two identical objects placed in distant areas of the open field (the sample phase). In the testing phase (i.e., in trial 3), one of the objects was moved to a new area (i.e., the object in a new place) while the other object remained in the previous place (i.e., the object in the familiar place). By employing a Noldus-Ethovision video-tracking system, trials 2 and 3 were video recorded to ascertain the amount of times spent with each of the two objects. The length of time a rat’s nose was 1 cm away from the marked object area determined the total exploration for each object. Only animals that explored objects for ≥9 seconds in trial 3 were included for data analysis. Application of this criterion resulted in 10-15% attrition in the number of animals included for final data analysis (n=17-18/group). The results, such as the percentage of time spent in exploring the object in novel place and the object in the familiar place as well as the total object exploration time in trial 3 were computed. The proportion of times spent with the object in novel place was calculated by using the following formula: the time spent with the object in novel place/the total object exploration time × 100.

### Novel object recognition test (NORT)

The test for each rat comprised three consecutive trials separated by 15-minute inter-trial intervals in an open field. The detailed procedure employed in this test is described elsewhere [44-45]. The first two trials comprised acclimatization to an open field for 5 minutes (trial 1) and exploration of two similar objects placed in two areas within the open field for 5 minutes (trial 2). In the testing phase (trial 3), one of the objects was replaced with a new object (novel object) while the other object remained in its location (familiar object). The trials 2 and 3 were video recorded using Noldus-Ethovision video-tracking system. Only animals that explored objects for ≥9 seconds in trial 3 were included for data analysis. Application of this criterion resulted in 6-20% attrition in the number of animals included for final data analysis (n=16-17/group). The proportion of times spent exploring the novel object and the familiar object, and the total object exploration times were computed in trial 3. The length of time a rat’s nose was 1 cm away from the marked object determined the total familiar object or novel object exploration time. The proportion of times spent with the novel object was calculated by using the following formula: the time spent with the novel object / the total object exploration time × 100.

### Pattern separation test (PST)

The test encompassed four consequent trials divided by 30-minute inter-trial intervals, as described in our previous report [45]. The animal was first familiarized to the open field apparatus for 5 minutes (trial 1). Next, each rat sequentially explored two distinctive sets of identical objects (object types 1 and 2) laid on diverse types of floor patterns (Patterns 1 and 2 [P1 and P2]) for 5 minutes each in the two acquisition trials (trial 2 and trial 3) separated by 30 minutes. In the testing phase (trial 4), the floor pattern employed in trial 2 (P2) was maintained, and each rat explored an object from trial 3 (which is now a familiar object on P2) and an object from trial 2 (which is now a novel object on P2). Noldus-Ethovision video-tracking system recorded trials 3-4. The length of time a rat’s nose was 1 cm away from the object area determined the total novel object or familiar object exploration time. Only animals that explored objects for ≥9 seconds in trial 3 were included for data analysis. Application of this criterion resulted in 18-30% attrition of total animals included for final data analysis in both groups (n=13-14/group). The results, such as the proportion of times spent in exploring the novel object on P2 and the familiar object on P2 and the total object exploration time, were computed from trial 4. Furthermore, novel object discrimination index was calculated by using the following formula: the time spent with the novel object on P2/the total object exploration time × 100.

### Sucrose preference test (SPT)

The test measured anhedonia or depressive-like behavior evident from a decreased preference for sweet fluids such as sucrose or saccharin solution [46-48]. First, each rat was housed individually and given free access to two identical bottles containing 1% sucrose solution to adapt to the sucrose solution for 24 hours (day 1). Then, one of the bottles was replaced with a new bottle containing regular water for 24 hours (day 2), followed by a complete deprivation of water and food for 23 hours (day 3). Next, the rat was given free access to two bottles, one containing 100 ml of sucrose solution and another containing 100 ml of regular water but no food (day 4). An hour later, consumption of fluid from both bottles was recorded. After the completion of the test, the animals were placed back to their previous housing conditions with continuous access to unlimited water and food. Sucrose preference rate was calculated using the formula, sucrose consumption/(water consumption+sucrose consumption) ×100.

### Brain tissue processing and immunohistochemical studies

Age-matched naïve controls and a cohort of CERs (at 5 months after SE) were deeply anesthetized and perfused with 4% paraformaldehyde. The brains were removed, post-fixed in 4% paraformaldehyde for ~14 hours, and processed for cryostat sectioning following cryoprotection with sucrose solution. Coronal sections (30-micrometer thick) through the entire hippocampus were collected serially in 24-well plates containing the phosphate buffer. Serial sections (every 15th or 20th) along the septotemporal axis of the hippocampus were selected and processed for immunohistochemistry, as described in our previous studies [35, 45, 49-51].

The sections were etched with 20% methanol and 3% hydrogen peroxide in PBS for 20 minutes, washed three times in PBS, incubated for 30 minutes in PBS containing 0.1% Triton-X 100 and an appropriate serum (10%) chosen depending on the species in which the secondary antibody was raised. Following 16-hour incubation in a respective primary antibody solution, the sections were washed thrice in PBS and incubated in an appropriate secondary antibody for 60 minutes. The primary antibodies employed included mouse anti-parvalbumin (anti-PV, Sigma P3088, 1:2000), rabbit anti-neuropeptide Y (anti-NPY, Peninsula Laboratories, T-4070, 1:10,000), goat anti-doublecortin (anti-DCX, Santa Cruz, sc-8066, 1:300), rabbit anti-glial fibrillary acidic protein (anti-GFAP, Millipore, MAB360, 1:1000), rabbit anti- Ionized calcium binding adaptor molecule 1 (anti-IBA-1, Abcam, AB5076, 1:1000) and mouse anti- zinc transporter 3 (anti-ZnT3, Synaptic systems, 197011, 1:250). The secondary antibodies comprised biotinylated anti-mouse, anti-rabbit or anti-goat antibodies from Vector Labs (BA-2001, BA-1000, BA-9500). The sections were washed thrice in PBS and treated with an avidin-biotin complex reagent (Vector Lab, PK-6100) for an hour. The peroxidase reaction was developed using diaminobenzidine (Vector Lab, SK-4100) or vector SG (Vector Lab, SK-4700) as chromogens. After a thorough wash, the sections were mounted on gelatin-coated slides, dehydrated, cleared and coverslipped with permount.

### Stereological quantification of cells and neurons

We employed a stereological method to quantify the numbers of (i) PV+ interneurons in the DG; ii) NPY+ interneurons in the DG; iii) DCX positive newly born neurons in the granule cell layer and subgranular zone (GCL-SGZ); and iv) IBA-1+ microglia in the different hippocampal cell layers, as detailed in our previous reports [32, 35, 45, 49]. The stereological method comprised the optical fractionator counting in the StereoInvestigator system (Microbrightfield Inc., Williston, VT). The StereoInvestigator program computed the total number of cells in each chosen region by utilizing the optical fractionator formula, as detailed in our earlier reports [32, 35, 49].

### Measurement of GFAP+ structures in the hippocampus

Area fractions of GFAP+ astrocyte elements in multiple regions of the hippocampus were measured, as described in our previous study [50]. This quantification was performed using Image J for Windows. Briefly, gray-scale images of different regions of the hippocampus were digitized using a 20X lens in a Nikon E600 microscope outfitted with a digital video camera attached to a computer. A binary image was created from each image by choosing a threshold value that retained all GFAP+ elements but no background. The area occupied by the GFAP+ structures in the binary image was then quantified through “Analyze” function in Image J. The area fraction of GFAP+ structures were calculated for the entire hippocampus by using data from all selected serial sections.

## RESULTS

### Induction of SE and mortality following graded KA injections in young F344 rats

Graded intraperitoneal injections of KA (5 mg/Kg/hour for 2-5 hours) to 6-8 weeks old male F344 rats induced robust SE in a vast majority of animals. Injection of KA was stopped once the rat started to show continuous Stage-IV or intermittent Stages III-V seizures for 10 minutes. Animals exhibiting such robust SE continued to have a similar pattern of seizures for over two hours. However, to minimize mortality and to obtain animals displaying SE for a comparable duration, convulsive seizures in all animals were terminated with a single subcutaneous injection of diazepam (10mg/Kg) 2 hours after the onset of SE. Nearly 20% of rats showed uncontrolled bouncing seizures and/or tonic-clonic seizures ~30-60 minutes after SE onset. The rats exhibiting bouncing or tonic-clonic seizures were restrained using towels, massaged thoroughly for a couple of minutes, and placed in a cage that lacked bedding but located on the top of another cage partially filled with ice flakes. These handling and hypothermia procedures were performed to facilitate muscle relaxation and to prevent tonic-clonic seizure-induced death. Out of 173 rats used for KA injections, 167 rats (~97%) developed robust SE. Among rats that developed robust SE, 13 rats (~8%) died before their scheduled diazepam injection. No mortality was observed after the administration of diazepam. Thus, the KA model described in this study is efficient for inducing robust SE with minimal death.

### Development of chronic epilepsy in the 3^rd^ month after SE

In the 3^rd^ month after SE, the presence of chronic epilepsy in all SE survivors (154 rats) was determined through scoring of behavioral SRS (convulsive seizures) from direct observations for 48 hours (6 hours/day for 8 days, total, 48 hours). All SE survivors developed chronic epilepsy, which was evident from the occurrence of repeated stages III-V seizures during the observation period. The average frequencies were 0.24 ± 0.01/hour for all SRS (i.e., ~6 seizures/day), and 0.2 ± 0.01/hour for stage-V SRS (i.e., ~5 stage-V seizures/day). The average duration of individual seizures was 36.75 ± 0.56 seconds, whereas, the total time spent in seizure activity for the recorded period was 0.24 ± 0.01 (%) (i.e., ~3.5 minutes/day). Among 154 rats, 126 rats (~82%) displayed a frequency of seizures in the range of 5-10 seizures/day, 16 rats (~10%) exhibited >10 seizures/day and 12 rats (~8%) presented <5 seizures/day. Thus, all rats that displayed robust SE showed chronic epilepsy in the 3^rd^ month after SE.

### The extent of SRS activity in CERs in the 5^th^ and 6^th^ month after SE

**Figure 1.**
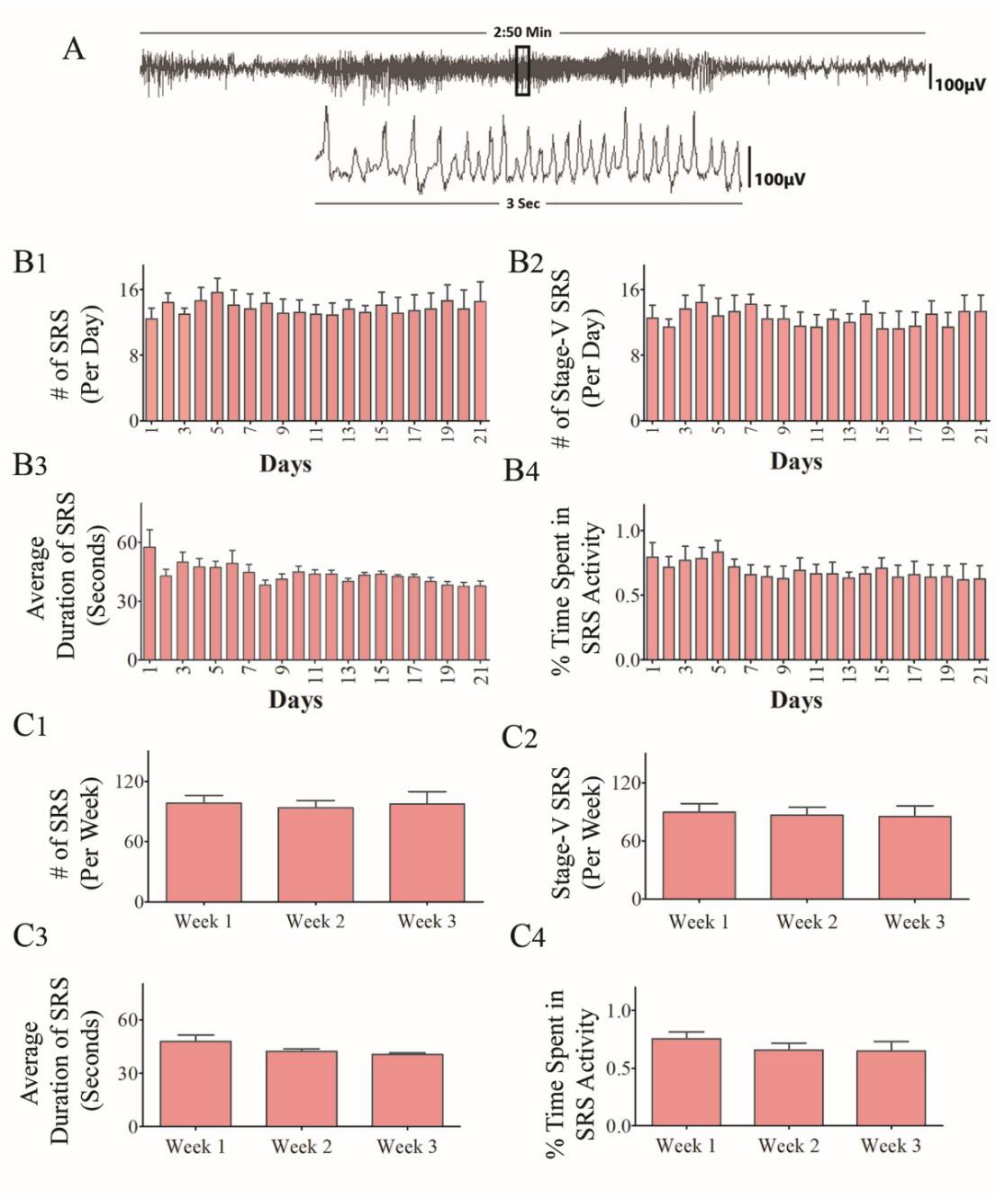
Analysis of time-locked video-EEG tracings from chronically epileptic rats (CERs) in the 5^th^ month after induction of status epilepticus (SE). Data from 3 weeks of continues EEG recordings are illustrated. (A) An example of EEG tracings during a spontaneous recurrent seizure (SRS). The bar charts in B1-B4 compare daily SRS activity occurring over 21 consecutive days. Note that, the number of SRS per day (B1), the number of stage-V SRS/day (B2), the duration of individual SRS (B3), and the percentage of time spent in SRS activity (B4) were comparable across 21 days (p>0.05, repeated measures ANOVA, RM-ANOVA). The bar charts in C1-C4 compare weekly SRS activity occurring over 3 consecutive weeks. Note that, the number of SRS per week (C1), the number of stage-V SRS/week (C2), the duration of individual SRS (C3), and the percentage of time spent in SRS activity (C4) were comparable across 3 weeks (p>0.05, RM-ANOVA).

**Figure 2.**
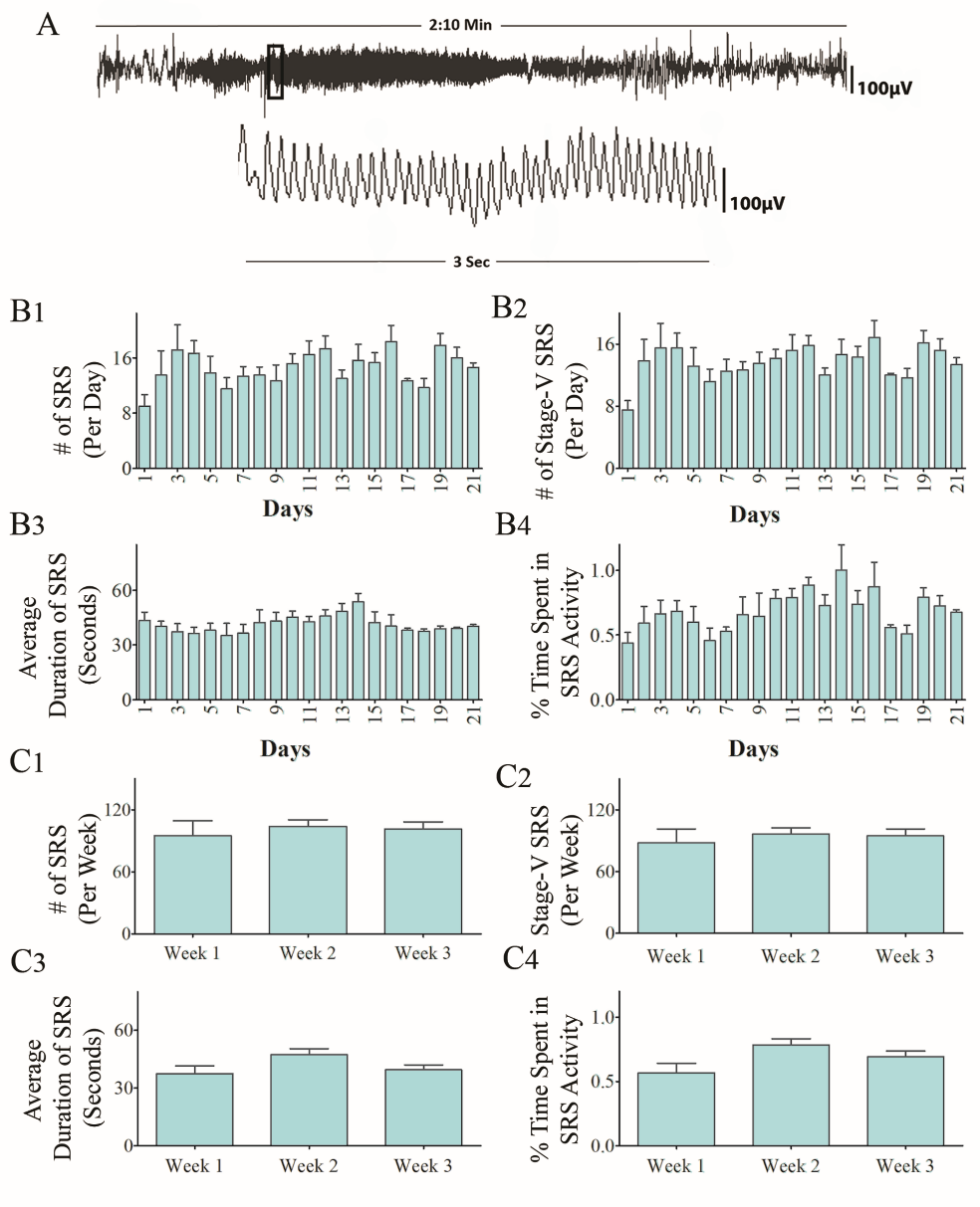
Analysis of time-locked video-EEG tracings from chronically epileptic rats (CERs) in the 6^th^ month after induction of status epilepticus (SE). Data from 3 weeks of continues EEG recordings are illustrated. (A) An example of EEG tracings during a spontaneous recurrent seizure (SRS). The bar charts in B1-B4 compare daily SRS activity occurring over 21 consecutive days. Note that, the number of SRS per day (B1), the number of stage-V SRS/day (B2), the duration of individual SRS (B3) , and the percentage of time spent in SRS activity (B4) were comparable across 21 days (p>0.05, repeated measures ANOVA, RM-ANOVA). The bar charts in C1-C4 compare weekly SRS activity occurring over 3 consecutive weeks. Note that, the number of SRS per week (C1), the number of stage-V SRS/week (C2), the duration of individual SRS (C3), and the percentage of time spent in SRS activity (C4) were comparable across 3 weeks (p>0.05, RM-ANOVA).

**Figure 3.**
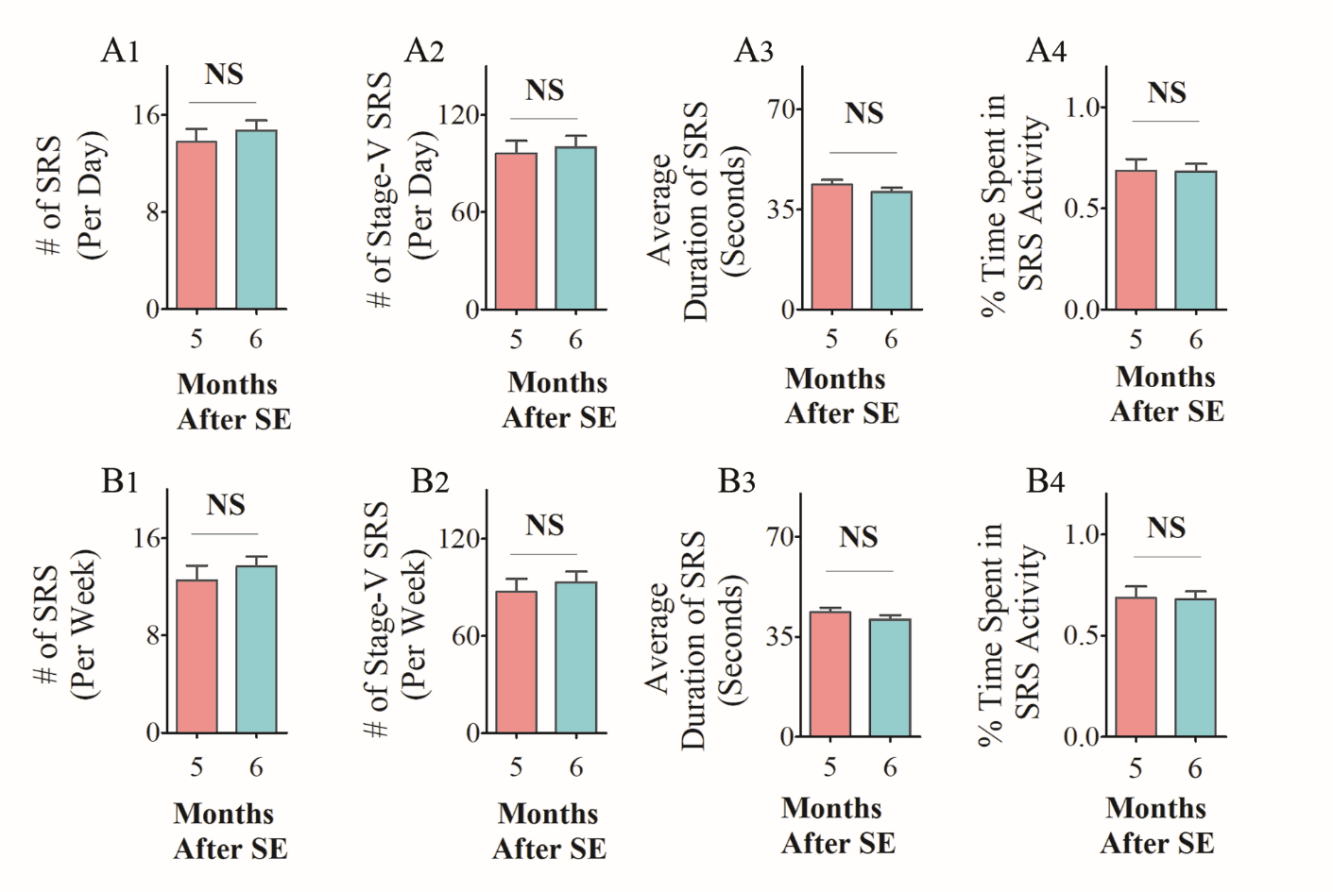
Comparison of EEG data taken from chronically epileptic rats (CERs) in the 5^th^ and 6^th^ month after status epilepticus (SE). The bar charts in A1-A4 compare the average daily SRS activity occurring over 21 consecutive days. Note that, the number of SRS per day (A1) , the average number of stage-V SRS/day (A2), the duration of individual SRS (A3), and the percentage of time spent in SRS activity (A4) were comparable between CERs recorded in the 5^th^ month after SE and CERs recorded in the 6^th^ month after SE (p>0.05, two-tailed, unpaired Student’s t-test). The bar charts in B1-B4 compare the average weekly SRS activity occurring over 3 weeks. Note that, the number of SRS per week (B1), the number of stage-V SRS per week (B2), the duration of individual SRS (B3), and the percentage of time spent in SRS activity (B4) were comparable across 3 weeks (p>0.05, two-tailed, unpaired Student’s t-test). NS, not significant.

To determine consistency in the frequency and intensity of SRS at extended time-points after SE, we randomly chose two cohorts of CERs and recorded continuous EEG activity for 21 days. The recordings were made in the 5th month after SE for one group and in the 6th month after SE for another cohort. Animals that lost electrodes during the period of video-EEG recordings were excluded from the study. Only CERs with 21 days of continuous video-EEG recordings were included for data analyses in the 5th month (n=9) and the 6th month (n=5) after SE. The EEG tracings in panel A of figures 1 and 2 illustrate the electrical activity and poly-spikes (>35 in 3 seconds) occurring during a spontaneous seizure (Fig. 1 and 2A). Both cohorts of CERs showed a similar frequency of all SRS, and stage-V SRS over 21 days (Fig. 1 and 2 B1-B2, p>0.05, repeated measures ANOVA). Furthermore, the average duration of individual SRS (Fig. 1 and 2B3, p>0.05, RM- ANOVA), as well as the percentages of time spent in SRS activity for the recording periods (Figs. 1 and 2B4, p>0.05, RM- ANOVA), were also comparable across 21 days. Analyses of numbers of all SRS, numbers of stage-V SRS, the average duration of individual SRS, and percentages of time spent in SRS activity were also similar when data were compared as the sum of SRS activity per week (Fig. 1 and 2C1-C4, p>0.05). Moreover, all parameters of EEG data between CERs measured in the 5th or 6th month after SE were similar when comparisons were made as an average data per day (Fig. 3A1-A4, p>0.05) or per week (Fig. 3B1-B4, p>0.05). Thus, robust and consistent SRS activity was observed in the 5th and 6th month after SE in the KA model detailed in this study.

### Frequency of SRS in the day-light and night periods in CERs

**Figure 4.**
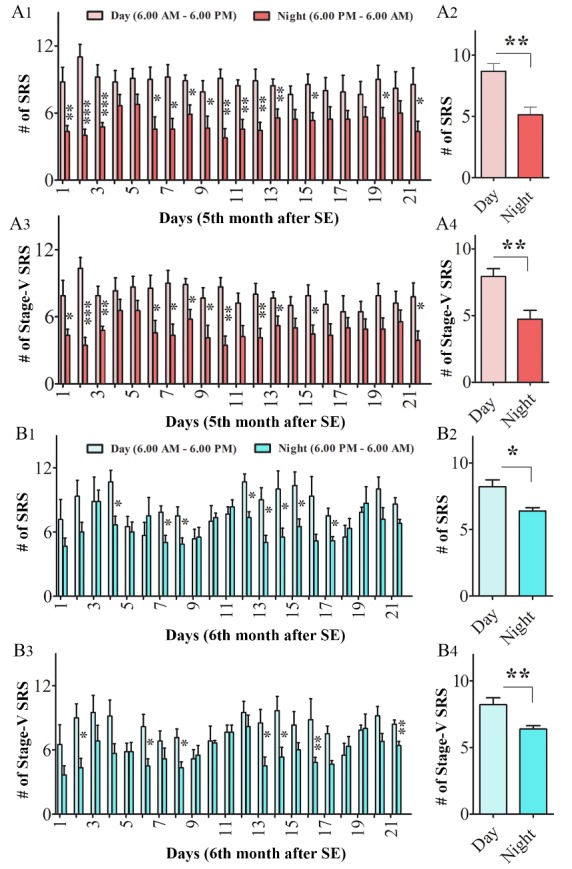
Comparison of daily EEG data from daylight (6 AM to 6 PM) and night (6 PM to 6 AM) periods in the 5^th^ and 6^th^ month after status epilepticus (SE). The bar charts in A1 and A3 illustrate consistently higher frequencies of SRS (A1) and Stage-V SRS (A3) in daylight periods than night periods in the 5^th^ month after SE. The bar charts in A2 and A4 compare the average frequency of SRS (A2) and Stage-V SRS (A4) over 21 consecutive days between the daylight and night periods in the 5^th^ month after SE. The bar charts in B1 and B3 illustrate higher frequencies of SRS (B1) and Stage-V SRS (B3) in daylight periods than night periods in the 6^th^ month after SE. The bar charts in B2 and B4 compare the average frequency of SRS (B2) and Stage-V SRS (B4) between the daylight and night periods in the 6^th^ month after SE. *, p< 0.05; **, p< 0.01; ***, p<0.001; NS, not significant.

To examine the circadian variations in SRS activity in this model, we analyzed the frequency and intensity of SRS during the daylight period (6.0 AM-6.0 PM) vis-à-vis the night or dark period (6.0 PM-6.0 AM) for the entire duration of 21 days of EEG recordings for both cohorts of CERs. This analysis showed a strong trend of reduced frequencies of SRS in the night period compared to the daylight period for each day of recordings performed for CERs in the 5th and 6th month after SE (Fig. 4A1-B4). In the 5th month after SE, the differences in SRS frequency between daylight and night periods were significant for days 1-3, 6-13, 15, 19 and 21 (Fig. 4A1, p<0.05 to 0.001). The differences in stage V-SRS frequency between daylight and night periods were significant for days 1-3, 6-10, 12-13, 15, and 21 (Fig. 4A3, p<0.05 to 0.001). A similar trend was observed when the numbers of all SRS and stage-V SRS recorded in the daylight and night periods during the entire 21 days were compared (Fig. 4A2, B2, p<0.01). Similar results were seen when recordings made in the 6th month after SE were compared across daylight and night periods. The differences in SRS frequency between daytime and night periods were significant for days 4, 7-8, 12-15, and 17 (Fig. 4B1, p<0.05). The differences in stage V-SRS frequency between daylight and night periods were significant for days 2, 6, 8, 13-14, 16, and 21 (Fig. 4B3, p<0.05 to 0.01). The findings were similar when the numbers of all SRS and stage-V SRS recorded during the entire 21 days were compared across daylight and night periods (Fig. 4B2, B4, p<0.05 to 0.01). Furthermore, there were no differences when frequencies of SRS occurring during daylight or night periods were separately compared across 21 days (p>0.05, RM-ANOVA), Thus, the rates of SRS in CERs were consistently higher in the daylight period, in comparison to the night or dark period, and frequencies of SRS occurring during both daylight and night periods were consistent over 21 days. Overall, the KA model described here showed an increased frequency of SRS in the daylight period.

**Figure 5.**
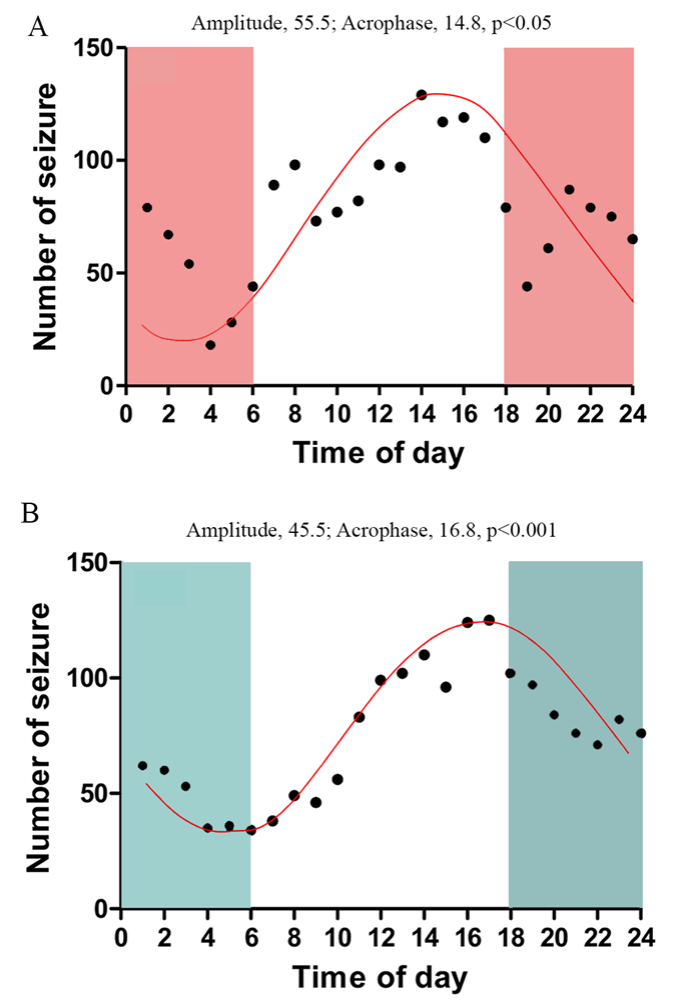
24-hour periodicity of seizures occurring in the 5^th^ month (A) and 6^th^ month (B) after status epilepticus (SE). In each month, EEG data from 21 consecutive days were used for plotting the periodicity of seizures. Note that, the SRS activity peaks during the daylight period and drops in the night period, in both the 5^th^ month (p<0.05) and 6^th^ month (p<0.001) after SE.

### 24-hour periodicity of SRS in CERs

Since frequencies of SRS occurring during both daylight and night periods were consistent over 21 days in both cohorts of CERs examined in the 5th and 6th month after SE, we examined 24-hour periodicity of seizures using >300 seizures occurring in each animal during the 21-days of video-EEG recordings. Acrophase analyses of summed seizures among all animals showed 24-hour periodicity of occurrence with an amplitude ranging from 45.5 to 55.5 and an acrophase ranging from 14.8 to 16.8 (Fig. 5A, B). As illustrated in the earlier section, the incidence of seizures was predominant during the daylight phase when the animals were less active, which was evident in both cohorts of CERs. Notably, the frequency of SRS activity peaked between 2-6 PM in both groups of CERs. This rhythmic pattern was also seen when each animal was analyzed separately, with a small variation in acrophase (10.9 to 17.3). Thus, 24-hour periodicity was consistently observed in the 5th and 6th month after SE in the KA model described in this study.

### Cognitive and mood impairments in CERs

Cognitive and mood impairments are the most conspicuous co-morbidities associated with chronic epilepsy. We examined KA administered rats with cognitive, memory, and mood function tasks in the 5th month after SE to discern the extent of dysfunction.

### Hippocampus-dependent cognitive dysfunction in CERs

**Figure 6.**
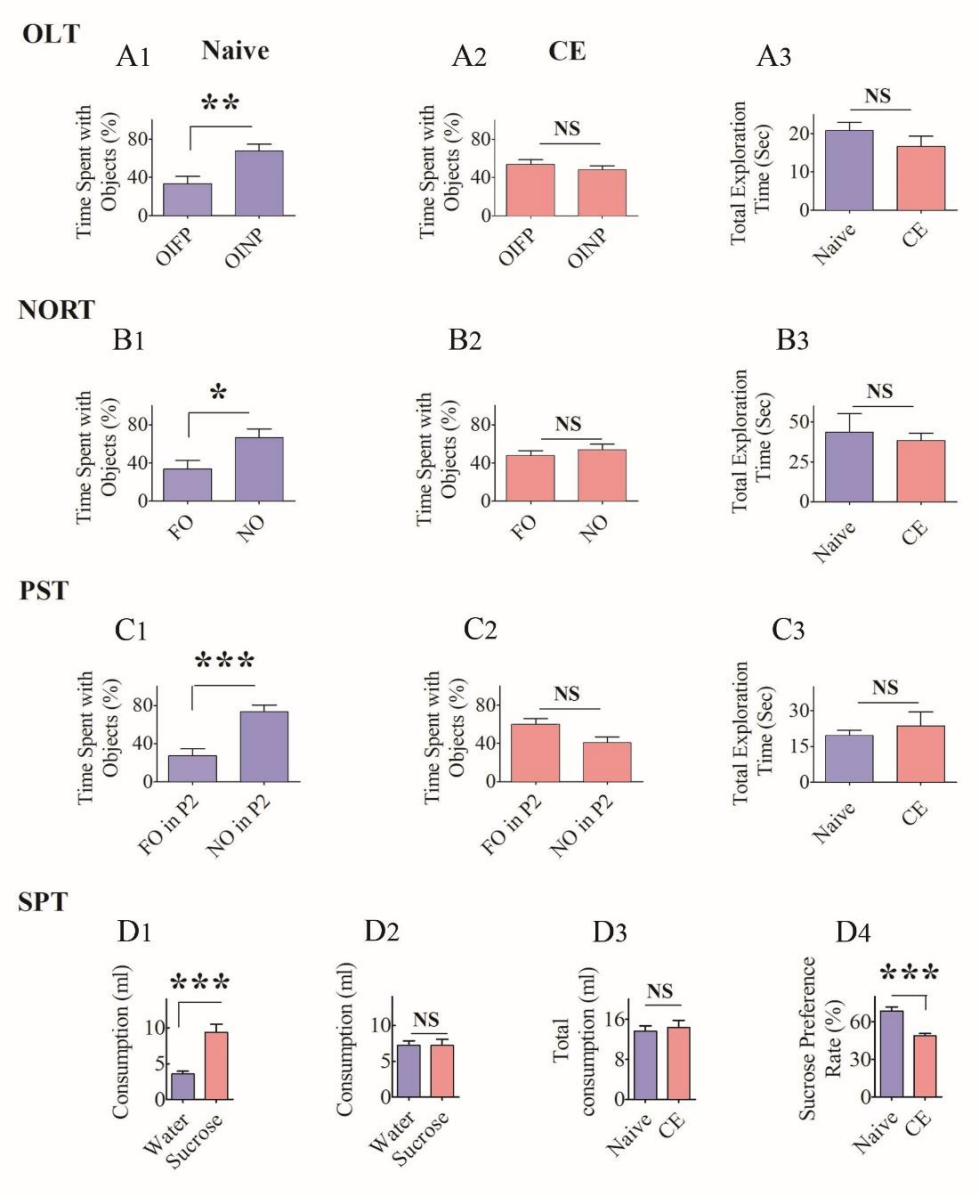
Chronically epileptic rats (CERs) displayed cognitive, memory, and mood impairments. The results of an object location test (OLT, A1-A3), a novel object recognition test (NORT, B1-B3), a pattern separation test (PST, C1-C3), and a sucrose preference test (SPT, D1-D4). The bar charts in A1-A2 compare percentages of time spent with the object in a familiar place (OIFP) and the object in a novel place (OINP) in naïve rats (A1) and CERs (A2) whereas, the bar chart in A3 compares the total object exploration time between naïve rats and CERs. Note that, naïve rats showed a higher propensity to explore OINP over OIFP (p<0.01) whereas, CERs showed no such preference in an OLT (p>0.05). The bar charts in B1-B2 compare percentages of time spent with the familiar object (FO) and the novel object (NO) in naïve rats (B1) and CERs (B2) whereas, the bar chart in B3 compares the total object exploration time between naïve rats and CERs. Note that, naïve rats showed higher propensity to explore NO over FO (p<0.05) whereas, CERs showed no such preference in a NORT (p>0.05). The bar charts in C1-C2 compare percentage of times spent with the familiar object on pattern 2 (FO of P2) and the novel object on pattern 2 (NO on P2) in naïve rats (C1) and CERs (C2) whereas, the bar chart in C3 compares the total object exploration time between naïve rats and CERs. Note that, naïve rats spent a greater amount of time with the NO on P2, in comparison to FO on P2 (p<0.001) whereas, CERs showed no such bias in a PST (p>0.05). The bar charts in D1-D2 compare consumption of regular water and sucrose-containing water in naïve rats (D1) and chronically epileptic rats (CERs). Note that, naïve rats preferred to drink sucrose-containing water (D1) whereas CERs showed no such preference (D2). The bar chart in D3 illustrates that the total consumption of fluid was comparable between naïve rats and CERs. The bar chart in D4 shows that the sucrose preference rate is greater in naïve rats in comparison to CERs (p<0.001). *, p< 0.05; **, p< 0.01; ***, p<0.001; NS, not significant.

We utilized an OLT, which examined the cognitive ability of animals to detect subtle changes in the immediate environment, a function linked to regular hippocampal network activity. After acclimatization and exploring a particular spatial arrangement of two identical objects in the sample phase, naïve control animals demonstrated cognitive ability to discern minor changes in the environment. The proficiency was evident from a significantly higher percentage of their object exploration time spent with the object moved to a novel place (OINP) than the object in a familiar place (OIFP) in the testing phase (p< 0.01, Fig. 6A1). In contrast, CERs showed impaired cognitive function by spending nearly equal amounts of their object exploration time with the OIFP and OINP (p>0.05, Fig. 6A2). The total object exploration time in the testing phase was comparable between naïve rats and CERs (p>0.05, Fig. 6A3), implying that the cognitive task was not influenced by differences in object exploration time by animals in the two groups. Thus, the KA model detailed in this study showed hippocampus-dependent cognitive impairment.

### Recognition memory dysfunction in CERs

We employed a NORT, which assessed the competence of animals for recognition memory, a function linked to regular neural activity in both the perirhinal cortex and the hippocampus. After familiarization to the open field apparatus in the habituation phase, and exploring two identical objects in the sample phase, naïve control rats showed proficiency for recognition memory by spending a significantly higher percentage of their object exploration time with the novel object (NO) than the familiar object (FO) in the testing phase (p< 0.05, Fig. 6B1). In contrast, CERs showed no preference for the NO, which was evident from the exploration of NO and FO for nearly equal amounts of time in the testing phase (p>0.05, Fig. 6B2). The memory task was not influenced by differences in object exploration time by animals in naïve and CER groups, as the total object exploration time was similar between the two groups of rats (p>0.05, Fig. 6B3). Thus, recognition memory dysfunction is one of the features of the KA model described in this study.

### Pattern separation impairment in CERs

We interrogated animals with a PST, which evaluated the competency of animals for pattern separation. Pattern separation function reflects the proficiency of animals for distinguishing similar but not identical experiences through storage of similar representations in a non-overlapping manner [52-53]. Following acclimatization in trial 1, and successively exploring two distinct sets of identical objects (object types 1 and 2) placed on separate types of floor patterns (P1 and P2) in the two acquisition trials (trials 2 and 3), naïve control rats showed excellent pattern separation ability. The proficiency of naïve rats was evident from their preference to explore the object from trial 2 (i.e., the novel object on pattern 2[NO on P2) than the object from trial 3 (i.e., the familiar object on pattern 2 FO on P2, p<0.001, Fig. 6C1). In divergence, CERs exhibited pattern separation dysfunction by exploring the FO on P2 and the NO on P2 in nearly equal proportions (p>0.05, Fig. 6C2). As in OLT and NORT, the pattern separation task was not influenced by differences in the object exploration time between naïve rats and CERs, as the total object exploration time was similar between the two groups of rats (p>0.05, Fig. 6C3). Thus, the KA model described in this study exhibited pattern separation dysfunction.

### Depressive-like behavior in CERs

Depression is a psychiatric symptom commonly seen in TLE patients. Anhedonia, a measure of depressive-like behavior, is typified by disinterest to engage in activities that are pleasurable when the mood function is normal. We investigated the extent of anhedonia in CERs through an SPT. After acclimatization to sucrose-containing water on day 1, both sucrose-containing water and regular water on day 2, and food and water deprivation on day 3, naïve control rats displayed normal mood function by consuming a higher volume of sucrose-containing water than the regular water on day 4 (p<0.01, Fig. 6D1). On the other hand, CERs consumed nearly equal amounts of sucrose-containing water and regular water (p>0.05, Fig. 6D2). The task was not influenced by differences in fluid consumption, as both naïve and CERs consumed nearly equal amounts of fluid (p>0.05, Fig. 6D3). The sucrose preference rate was also higher in naïve control rats than CERs (p<0.001, Fig. 6D4). Thus, CERs in this KA model displayed depressive-like behavior.

**Figure 7.**
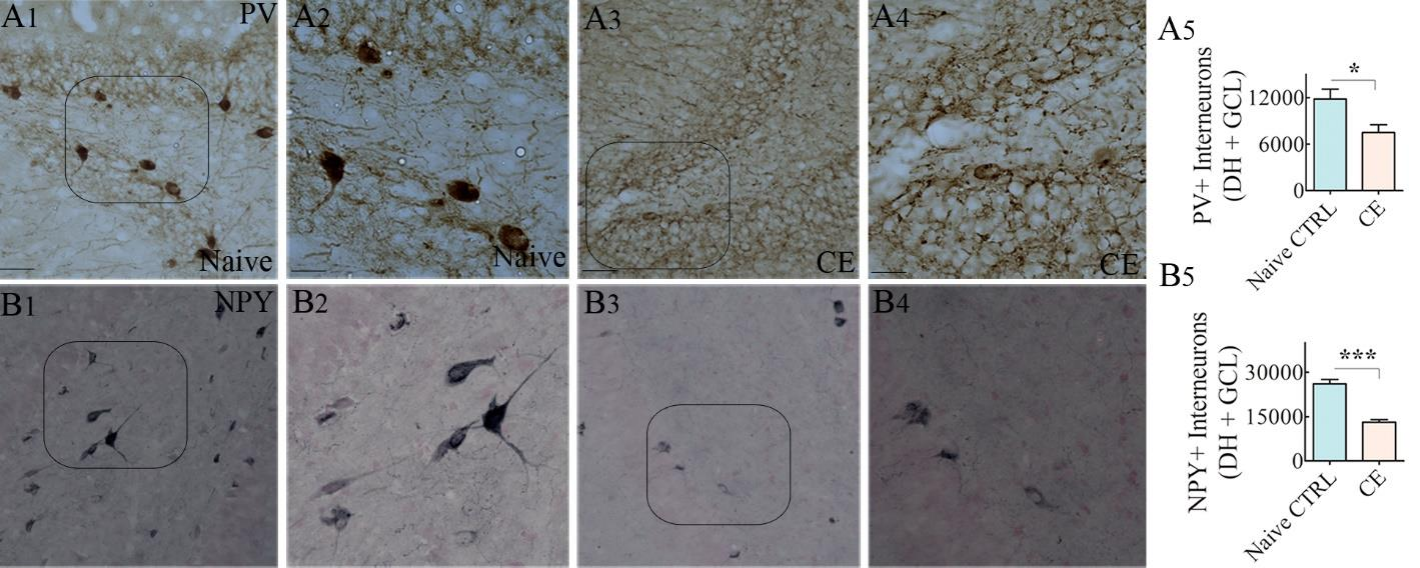
Chronically epileptic rats (CERs) demonstrated reduced numbers of interneurons positive for parvalbumin (PV) and neuropeptide Y (NPY) in the dentate gyrus (DG). (A1-A4) illustrate the distribution of PV+ interneurons in a naïve control DG (A1) and a CER (A3). (A2) and (A4) are magnified views of regions from A1 and B1. (B1-B4) illustrate the distribution of PV+ interneurons in the DG of a naïve control rat (B1) and a CER (B3). (B2) and (B4) are magnified views of regions from B1 and B3. The bar charts show that interneurons positive for PV (A5) and NPY (B5) are reduced in number in the DG of CERs. *, p<0.05; ***, p<0.001. Scale bar, A1, A3, B1 and B3, 50 μm; A2, A4, B2 and B4, 25 μm.

### Loss of GABA-ergic interneurons expressing PV and NPY in CERs

**Figure 8.**
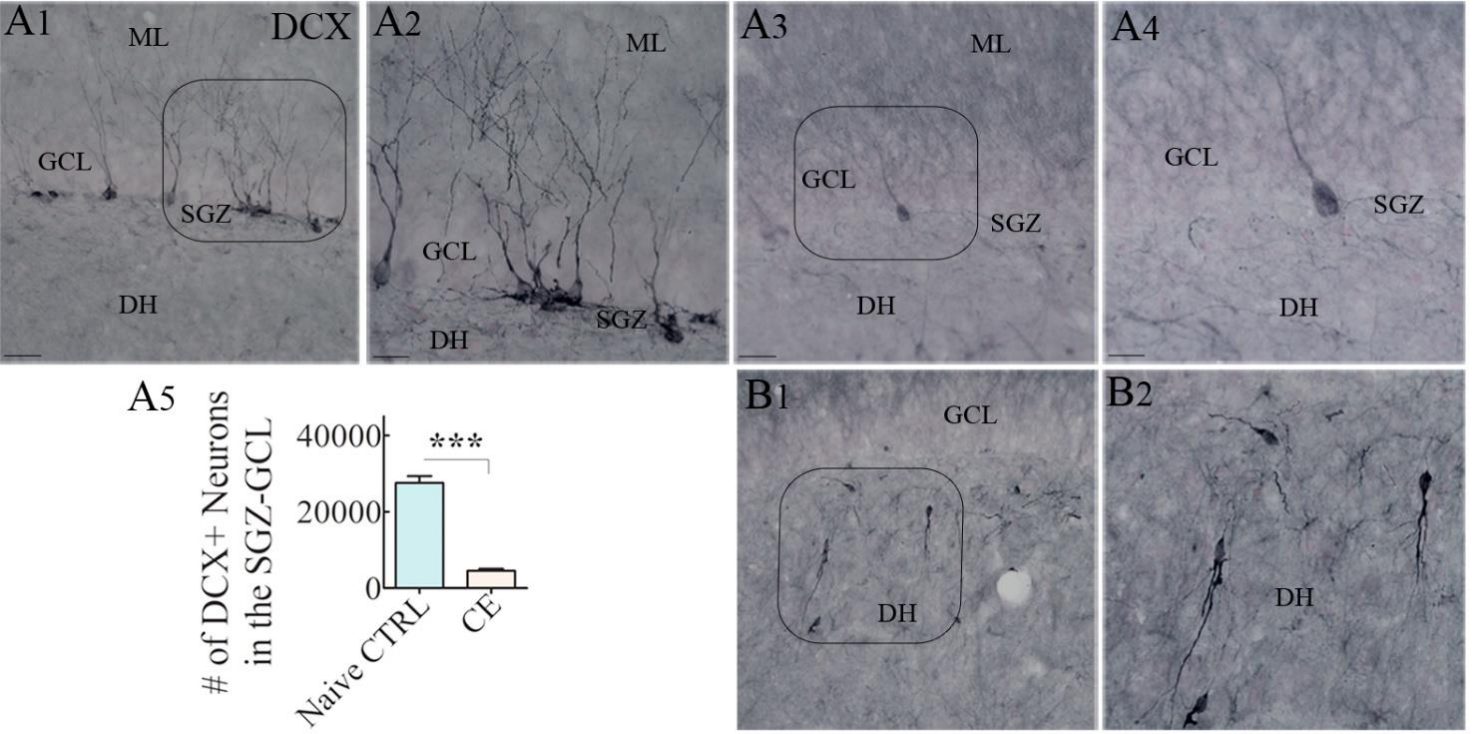
Chronically epileptic rats (CERs) demonstrated reduced neurogenesis in the dentate subgranular zone-granule cell layer (SGZ-GCL). (A1-A4) illustrate the distribution of doublecortin-positive (DCX+) newly born neurons from a naïve control rat (A1) and a CER (A3). A2 and A4 are magnified views of regions from (A1) and (A3). The bar chart in (A5) shows that the number of newly born DCX+ neurons is reduced in the hippocampus of CERs. (B1) illustrates the abnormal migration of newly born DCX+ neurons into the dentate hilus. (B2) is a magnified of a region from B1. ***, p<0.001. Scale bar, A1, A3 and B1, 50 μm; A2, A4 and B2, 25 μm.

We examined GABA-ergic interneurons expressing PV and NPY using immunohistochemical staining of serial sections through the hippocampus from naïve rats and CERs (Fig. 7A1-B4, n=11-12/group). In comparison to naïve control rats (Fig. 7A1-A2, B1-B2), densities of both PV+ and NPY+ interneurons appeared to be reduced considerably in the DG of CERs (Fig. 8A3-A4, B3-B4). Stereological quantification confirmed that the numbers of PV+ and NPY+ interneurons in the DG of CERs were significantly reduced in CERs, in comparison to naïve rats (Fig. 7A5, B5). Thus, CERs in this KA model displayed reduced numbers of interneurons in the DG, which is consistent with the results reported for previous KA models [22-24, 46, 47].

### Changes in hippocampal neurogenesis in CERs

We investigated newly born neurons expressing DCX using immunohistochemical staining of serial sections through the hippocampus from naïve rats and CERs (Fig. 8A1-A4, n=10-11/group). The population of newly born neurons expressing DCX appeared to be diminished drastically in the SGZ-GCL of CERs. Stereological quantification confirmed that the numbers of DCX+ newly born neurons were considerably reduced in CERs, in comparison to naïve animals (Fig. 8A5). Furthermore, a significant amount of aberrant neurogenesis was observed in CERs, which is typified by the presence of abnormally migrated newly born DCX+ neurons in the dentate hilus (Fig. 8B1, B2), in comparison to naïve rats showing no or occasional newly born neurons in the dentate hilus. Thus, CERs in this KA model displayed reduced hippocampal neurogenesis, which is consistent with findings in previous KA models [32, 39].

### Neuroinflammation in the hippocampus of CERs

**Figure 9.**
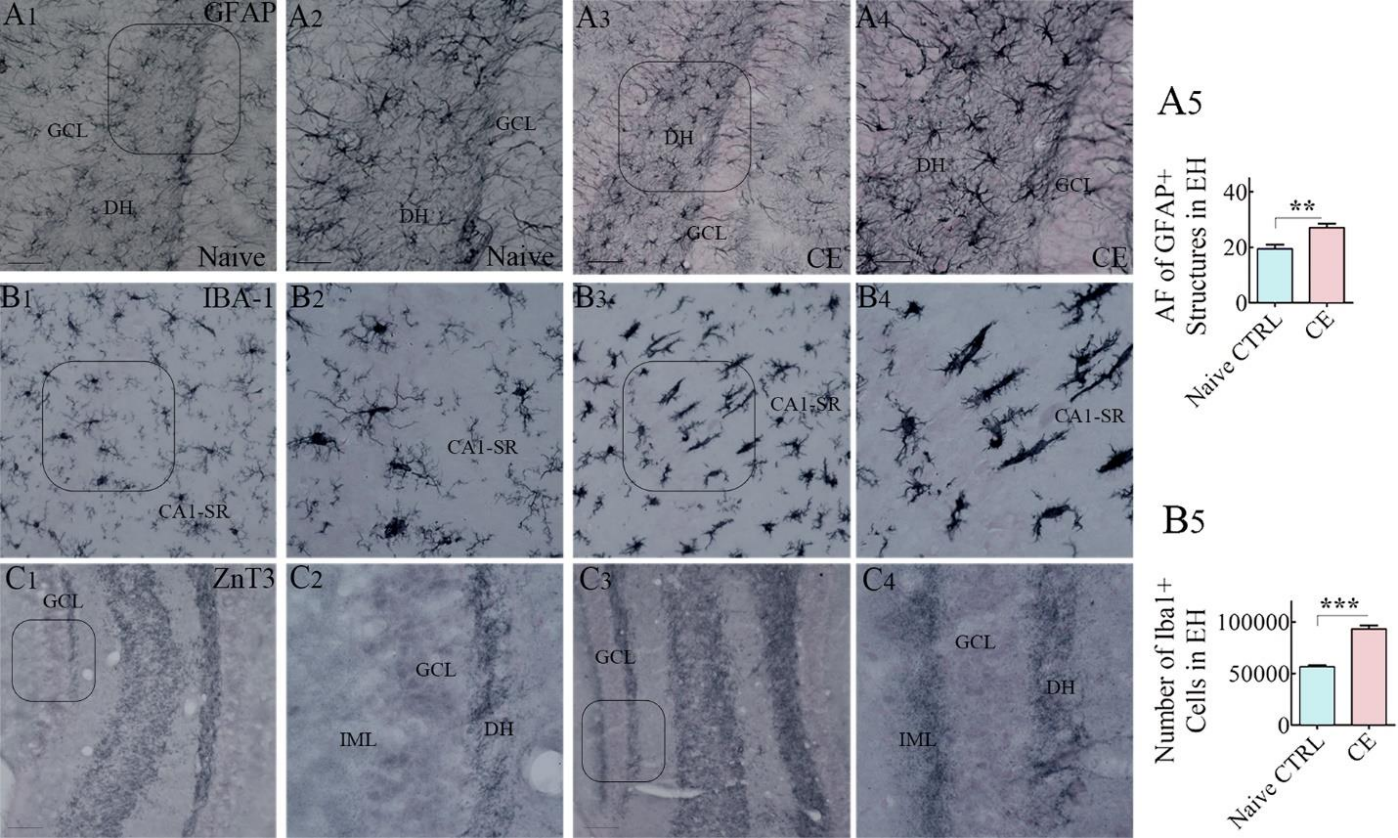
Chronically epileptic rats (CERs) showed hypertrophy of astrocytes, activated microglia, and abnormal sprouting of mossy fibers in the hippocampus. A1-A4) illustrate the distribution of glial-fibrillary acidic protein-positive (GFAP+) astrocytes in the dentate gyrus (DG) of a naïve control rat (A1) and a CER (A3). A2) and B4) are magnified views of regions from A1 and A3. The bar chart in A5 shows that the area fraction of GFAP+ elements is increased in the entire hippocampus (EH) of CERs. Figures B1-B4 illustrate the distribution of IBA-1 positive microglia in the CA1 subfield of a naïve control rat (B1), and a CER (B3). B2) and B4) are magnified views of regions from B1 and B3. Note that, the CA1 stratum radiatum in the CER displays rod-shaped activated microglia. The bar chart in A5 shows that the number of IBA-1+ microglia is increased in the entire hippocampus (EH) of CERs. C1-C4) illustrate the distribution of ZnT3+ positive mossy fibers in the DG of a naïve control rat (C1), and a CER (C3). (C2) and (C4) are magnified views of regions from C1 and C3. Note abnormal sprouting of mossy fibers into the inner molecular layer (IML) of the DG in the CER. **, p<0.01; ***, p<0.001. Scale bar, A1, A3, B1, B3, C1 and C3, 50 μm; A2, A4, B2, B4, C2 and C4, 25 μm.

The extent of neuroinflammation in the hippocampus of CERs was examined through immunohistochemical staining of serial sections from naïve rats and CERs for GFAP (a marker of astrocytes) and IBA-1 (a marker of microglia) (Fig. 9A1-A4, B1-B4, n=5-6/group). Immunostaining for GFAP showed hypertrophy of astrocytes in CERs, which was conspicuous in the dentate hilus (Fig. 9A3-A4), in comparison to naïve rats (Fig. 9A1-A2). Quantification of the area fraction of GFAP+ structures revealed that, in contrast to the naïve control group, CERs displayed increased density of hypertrophied astrocytes in the hippocampus (p<0.01, Fig. 9A5). Immunostaining for IBA-1 in naïve control rats revealed a uniform distribution of highly ramified microglia with relatively smaller soma. Such morphology was seen in all regions of the hippocampus (Fig. 9B1-B2]). However, the hippocampus of CERs displayed activated microglia, which showed hypertrophy of soma and processes, clustering of microglia or rod-shaped microglia (Fig. 9B3-B4). The rod microglia, exhibiting rod-like cell body with no polarized processes, were conspicuous in the CA1 subfield, an area of the hippocampus that shows consistent neurodegeneration in virtually all SE models [16-17, 20-21, 35, 55-56]. In CERs, multiple rod microglia were found to be aligned along their elongated axis in the CA1 stratum radiatum, perpendicular to the stratum pyramidale (Fig. 9B3-B4). Stereological quantification of microglia expressing IBA-1 in all cell layers of the hippocampus revealed increased numbers of microglia in CERs (Fig. 9B5). Thus, CERs in this KA model displayed chronic neuroinflammation in the hippocampus, which was evident from the presence of hypertrophied astrocytes, increased numbers of microglia, and the occurrence of activated microglia with rod-shaped soma.

### Aberrant mossy fiber sprouting in the DG of CERs

Both TLE patients and animals that experienced SE have consistently shown abnormal sprouting of axons of dentate granule cells into the inner molecular layer of the DG, a process known as aberrant mossy fiber synaptic reorganization. Such sprouting of mossy fibers increases the connectivity between granule cells and may increase the propensity for generating SRS [57-61]. We examined the occurrence of mossy fiber sprouting using ZnT3 immunostaining, which demonstrated the presence of aberrant mossy fiber sprouting in the dentate supragranular layer of CERs (Fig. 9C3-C4, in comparison to naïve control rats exhibiting no such sprouting (Fig. 9C1-C2). The extent of aberrant mossy fiber sprouting was generally more prominent in the lower blade of the GCL than the upper blade (Fig. 9C3). Thus, CERs in this KA model displayed a considerable amount of aberrant mossy fiber sprouting, which is also a feature of CERs in previous KA models (19, 21, 35, 61-62].

## DISCUSSION

Animal prototypes that mimic the most salient features of human TLE are necessary for reliably assessing the efficacy of promising new therapeutic agents or approaches. Multiple strategies have been proposed and investigated for preventing or treating epilepsy [45, 56, 63-72]. An essential prerequisite for succeeding in therapy development is the availability of animal prototypes with high predictive validity [73]. Therefore, rigorous investigation of novel strategies for TLE needs animal models that offer consistency for critical measures such as frequency and intensity of SRS over days, weeks and months, and cognitive and mood impairments. Besides, the prototype needs to display multiple neuropathological features of chronic TLE, including the loss of GABA-ergic interneurons, persistently waned and abnormal neurogenesis, chronic neuroinflammation, and aberrant mossy fiber sprouting. Numerous studies on SE and TLE have utilized a variety of mouse and rat models exhibiting different frequencies of SRS, behavioral co-morbidities and variable levels of neuropathology in the hippocampus and/or other regions of the limbic system [73-78]. Nonetheless, the epilepsy field is yet to identify a prototype that presents robust and consistent SRS over days, weeks, and months in the chronic phase of TLE.

Measurement of SRS using continuous video-EEG recordings for several weeks is considered a gold standard for detecting a model with a consistent or variable frequency of SRS. For instance, a previous study using continuous EEG recordings has discovered that the incidence of convulsive seizures regressed with time after KA-induced SE in C57BL/6J mice [43, 79]. Another study, by inducing SE with a single dose of KA at 10 mg/Kg demonstrated inconsistent chronic epilepsy development, exemplified by only 26% of animals exhibiting >4 seizures/day and the remaining 76% displaying ~0.5 SRS/day [40]. Moreover, many post-SE prototypes have been employed previously in therapeutic efficacy studies once the occurrence of one or a few SRS was recorded [80-83]. Such investigations are valuable for ascertaining the beneficial effects of promising therapeutic compounds or biologics with an early intervention approach (i.e., application of therapy occurring immediately after the diagnosis of epilepsy or when the epileptogenic processes are still evolving). However, for developing treatments for chronic TLE, promising therapeutic agents need to be examined when the chronically epileptic animals are exhibiting robust and consistent SRS.

Furthermore, many TLE models investigated in therapeutic studies have also been reported to have much lower (1-2 seizures/day) and inconsistent frequency of seizures, or clusters of SRS occurring one or more times a month [84-89]. The use of chronic epilepsy models with irregular frequency for SRS may lead to confounding results and interpretation of the therapeutic efficacy of drugs or biologics unless the study employs continuous video-EEG recordings for several months. Such prolonged EEG recordings are typically difficult to achieve for reasons such as the cost and the stability or malfunction of implanted electrodes over time in freely moving animals. By using graded injections of KA in young (6-8 weeks old) male F344 rats, a rat strain that is more susceptible for developing SE [90] and an age that exhibits minimal mortality despite presenting robust SE, we successfully produced a prototype where CERs displayed robust and consistent SRS. Both vigor and dependability of seizures were palpable from the occurrence of over 11 seizure episodes every day for 21 days in the 5th and 6th month after SE. These features make this model ideal for testing therapies in chronic epileptic conditions. Additional testing with commonly used antiepileptic drugs may further validate this prototype for studying drug-resistant epilepsy. Further investigations are required to determine whether induction of similar SE in older (>8 weeks old) male F344 rats or female rats of different ages would also result in a comparable chronic epileptic state.

The KA model presented in this study displayed three major phases, which include the SE phase, an intermediate phase, and the chronic epilepsy phase with robust and consistent frequency of SRS. Over 95% of animals developed robust SE after 2-5 graded injections of KA (SE phase). Upon termination of convulsive SE with a single dose of diazepam, animals advanced to a transitional period, which lasted ~2 months and during which animals exhibited occasional or inconsistent frequency of SRS (data not quantified). In the epilepsy literature, this phase is often referred to as “latent phase” showing no SRS. However, the purported “latent phase” has been challenged by many epilepsy investigators as animals experiencing SE typically display some SRS in the early period after SE, although the occurrences of such SRS are inconsistent in their frequency over weeks and months [41]. Some studies have also implied that epileptogenesis after a brain insult is progressive with continuing changes in the neuronal network extending into the chronic epilepsy period [91-93]. The current study did not investigate the occurrence of first SRS after SE as SRS were seen occasionally throughout the first two-month period after SE. Measurement of convulsive seizures in the 3rd month after SE through direct observations demonstrated that all SE survivors developed chronic epilepsy, though the frequency of SRS varied from 5-10 per day (82%), >10/day (10%) or <5/day (8%). Continuous video-EEG recordings performed in the 3rd and 4th month after SE demonstrated SRS in all animals but some animals displayed intermittent SRS (i.e., SRS events separated by several days of no SRS activity (data not illustrated). Variable frequencies of SRS confirmed that epileptogenic processes were still evolving in the 3rd and 4th month after SE in this model. Therefore, to identify a time-point at which animals that experienced SE displayed robust and unremitting frequency of SRS, we performed continuous video-EEG recordings for 21 days in the 5th and 6th month after SE. Two distinct groups of CERs, randomly chosen from a larger cohort, were utilized to ascertain continuity in the pattern of SRS frequency beyond the 5^th^ month after SE. In both of these cohorts, we found an unswerving frequency of SRS, which was evident when SRS were computed as events per day or week over three weeks.

A refined KA model developed in this study is unique because it exhibited the most salient features required for studying chronic TLE as well as mimicking several features of drug-resistant TLE. These comprise a high frequency of SRS unfailingly happening over days, weeks, and months with cognitive, memory, and mood impairments. Moreover, these behavioral alterations were associated with many neuropathological features of TLE, which include partial loss of GABA-ergic interneurons, significantly waned neurogenesis with persistent abnormal migration of a portion of newly born neurons, incessant neuroinflammation, and the occurrence of significant aberrant mossy fiber sprouting. The most compelling features that set this model apart from the previous KA and pilocarpine models in mice and rats are the robust frequency and consistency of SRS in the chronic phase (i.e., in the 5^th^ and 6^th^ month after SE) and a low (~8%) mortality during SE. Besides, CERs in these cohorts displayed SRS in a consistent circadian rhythm with a higher number of seizures occurring in the day-light period than the night or dark period. Such circadian rhythm of SRS in this model is also ideal for testing chronotherapy for TLE [94].

The animal prototype exhibited impairment in all cognitive or memory tasks and mood function investigated in this study. A weakened hippocampus-dependent cognitive function was evident from the inability of CERs to discern minor changes in the environment in an OLT. This test requires the hippocampus for encoding, consolidation, and retrieval [95-97] and is particularly sensitive to changes in the dorsal CA1 region [98]. As dorsal CA1 is one of the most vulnerable areas for exhibiting neurodegeneration following KA or pilocarpine-induced SE (35, 55-56], object location memory impairment in CERs is not surprising. The model also presented impaired recognition memory, which could be detected from a lack of proficiency of CERs to prefer novel objects over familiar objects in a NORT. This test requires normal network activity in the perirhinal cortex and the hippocampus to evaluate a previously encountered item as familiar [99]. The cognitive and memory impairments likely reflect the presence of abnormal network activity, in addition to ictal and inter-ictal events because, a considerable loss of PV+ and NPY+ interneurons, as observed in this model, can interfere with the type of network activity required for cognitive and memory tasks. For example, optimal operation of PV+ interneurons is critical for maintaining the working memory function of the hippocampus, as selective ablation of PV+ interneurons in the CA1 subfield induced spatial working memory impairments [100]. Diminished PV+ interneuron numbers can also be a crucial factor in the epileptogenic process, as a reduced number of synapses by PV+ interneurons can affect network properties and increase the susceptibility for developing seizures [100]. Likewise, NPY deficiency occurring from a partial loss of NPY+ interneurons, as observed in this model can contribute to learning, memory, and mood impairments. Such possibility is based on the role of NPY in the proliferation of hippocampal neural stem cells [101] and controlling excitatory neurotransmission [102] in addition to its anticonvulsant activity.

Furthermore, CERs generated in this study displayed an inability for pattern separation, which is a function to distinguish similar but not identical experiences [52-53]. Pattern separation is a competence that avoids confusion between similar memories by transforming similar input patterns of neural activity into dissimilar output patterns before their long-term storage in the hippocampus [103]. Since pattern separation function requires maintenance of DG neurogenesis at certain levels [104-105] and the integrity of DG circuitry [106], the results pointed to abnormalities in neurogenesis and alterations in DG network activity. Indeed, neurogenesis was substantially reduced, and a portion of newly born neurons continued to migrate into the dentate hilus in CERs, a feature also noted in previous KA models [32, 39]. Also, an abnormal synaptic reorganization was observed in the form of aberrant mossy fiber sprouting into the dentate inner molecular layer, which is a form of plasticity that increases connectivity between dentate granule cells, and likely also contributes to persistent hyperexcitability in the DG [57, 59-60]. Also, CERs generated in this study exhibited anhedonia, which is a symptom of depression where activities that bring pleasure in normal conditions are no longer pleasurable (i.e., hyposensitivity to happiness). Thus, a consistent frequency of robust SRS associated with behavioral co-morbidities is typically seen in chronic TLE, particularly drug-resistant TLE [107-109].

The chronic TLE model described here also demonstrated significant neuroinflammation, which was evident from a higher density of hypertrophied astrocytes (i.e., reactive astrocytes) and activated microglia in all subfields of the hippocampus. Most microglia in CERs exhibited an activated phenotype, typified by either hypertrophied soma with less or no ramified processes in DG and CA3 subfields, or rod-shaped microglia in the CA1 subfield. Rod microglia, first discovered in general paresis patients, represent a unique type of activated microglia with a sausage-shaped soma lacking polarized processes [110-112]. Rod microglia, also seen in the brain of patients with neurodegenerative disorders [113], express higher levels of MHC class II antigen [111]. The function of rod microglia is yet to be discerned. However, it has been suggested that the occurrence of rod microglia reflects an area of the brain with a milder to moderate injury but maintaining the integrity of the tissue [112]. Furthermore, rod microglia are likely involved in splinting damaged neuronal dendrites and axons, synapse stripping, and establishing a barricade to guard uninjured neurons or neuronal processes from a hostile milieu [110]. While activated microglia have been consistently observed in several models of chronic epilepsy, this is the first study to demonstrate the occurrence of rod microglia in the hippocampus of CERs exhibiting a robust and consistent frequency of SRS. A few previous studies have reported rod microglia in the dentate hilus at 1-5 days post-SE [114] and in the CA1 region at two weeks after SE [115]. Importantly, rod microglia have been observed in human brain samples associated with drug-resistant epilepsy [116], where they seemed to wrap apical dendrites of cortical neurons. Thus, it is likely that the occurrence of rod microglia in the CA1 subfield, particularly in the white matter region of stratum radiatum implies constant dendritic plasticity in CA1 pyramidal neurons with robust and consistent frequency of SRS, which may also be one of the significant features of drug-resistant epilepsy.

### Conclusions

This study presents a refined KA model of chronic TLE using 6-8 weeks old male F344 rats. A rigorous characterization in the chronic epilepsy period validated that the animal prototype mimics the most prominent characteristics of human TLE. The behavioral features include a constant frequency and intensity of SRS over days, weeks and months, and cognitive and mood impairments. Besides, the model displayed many neuropathological features that are typical to chronic TLE, which comprises a partial loss of GABA-ergic interneurons, waned and abnormal neurogenesis, chronic neuroinflammation, and aberrant mossy fiber sprouting. We propose that this consistent seizure model is ideal for investigating various antiepileptic therapies as well as comprehending multiple pathophysiological mechanisms associated with chronic TLE. It may be argued that a treatment that significantly reduces SRS in a model that exhibits robust and consistent frequency of SRS would likely be more successful in epileptic patients. Because of the consistency in the frequency and intensity of SRS over days, weeks and months with co-morbidities, the prototype mimics the features of pharmaco-resistant TLE. From this perspective, further testing in the chronic phase with the commonly used antiepileptic drugs may further validate this prototype for studying drug-resistant epilepsy.
